# Social Media Responses to the Pandemic: What Makes a Coronavirus Meme Creative

**DOI:** 10.3389/fpsyg.2021.569987

**Published:** 2021-03-08

**Authors:** Vlad Petre Glǎveanu, Constance de Saint Laurent

**Affiliations:** ^1^Department of Psychology and Counselling, Webster University Geneva, Bellevue, Switzerland; ^2^Centre for the Science of Learning and Technology, University of Bergen, Bergen, Norway; ^3^Department of Information Engineering, University of Bologna, Bologna, Italy; ^4^London School of Economics and Political Science, London, United Kingdom

**Keywords:** coronavirus, memes, social media, creativity, Reddit, creative process

## Abstract

The current pandemic and the measures taken to address it, on a global scale, are unprecedented. Times of crisis call for creative solutions, and these are not reduced to the work of scientists or politicians. In everyday life, both in online and offline spaces, people use their creativity to make sense of the current situation, to cope with it, and to learn its lessons. Social media is a privileged space for mundane and participative creativity through the production and sharing of coronavirus Internet memes. In this article, we examine the creativity of such memes from a dedicated Reddit community. We ask, in particular, what makes a coronavirus meme creative and what this creativity tells us about the pandemic and popular understandings of it. To answer these questions, we use a triangulation of quantitative and qualitative methods by having 480 memes coded by three social media users for surprise, meaningfulness, elaboration, humor, and creativity and qualitatively analyzing those memes that score highly on each dimension. An interesting finding concerns the importance of elaboration and humor for the evaluation of creativity in the case of memes, above the more traditional criteria of surprise (proxy for novelty) and meaningfulness (proxy for appropriateness), perhaps a feature unique for Internet spaces. The article ends with reflections on what these findings tell us about creativity on social media more generally and the creative processes involved in the generation and reception of coronavirus memes in particular.

## What Makes A Coronavirus Meme Creative

The COVID-19 pandemic poses a serious challenge for individuals and societies worldwide. Beyond the number of deaths and the impact on jobs and the economy, the measures imposed to stop the spread of the virus are difficult to cope with on a personal level. Being in lockdown or imposed social distancing disturbs daily life and calls for new and creative ways to make sense of the situation and adapt to it. In this context, social media becomes instrumental for connecting with others, sharing one’s experience, and generating new ways of understanding the experience of others. This is a rich medium, thus, for creativity researchers who aim to explore creative responses to the pandemic. In this article, we focus specifically on coronavirus Internet memes—cultural units, typically humorous in nature, circulating widely in digital environments and carrying meanings about the various facets of the epidemic, from the virus itself and its spread to protective equipment, measures, changes to everyday life, and broader economic and social impacts.

Outside of the current situation, social media is an intrinsically interesting domain of study for creativity researchers, especially those concerned by the social and cultural dynamic of this phenomenon. “As more of our world moves into online spaces, social media platforms become a central fountainhead for dispersed communities to share innovative ideas and original artifacts, as well as contribute to the discussions around those ideas” (Peppler and Solomou, 2011, p. 12). While the degree of novelty, originality, and meaningfulness of what is shared on social media will vary, it is precisely the act of *sharing*, which leads to the adaptation and transformation of content and ideas, that should concern us. Online creativity, as we discuss in this paper, is its own domain with a unique set of characteristics and consequences for individuals and society. Some of the latter are positive, for example being able to generate new ideas and build community and a sense of conviviality (Varis and Blommaert, 2015); others are distinctively negative. For instance,

“we are seeing how in many ways the internet has become as much about interaction with others as it has about accessing information. (⋯) In the drift from blogging, to social networking, to microblogging we see a shift from dialogue and communication between actors in a network, where the point of the network was to facilitate an exchange of substantive content, to a situation where the maintenance of a network itself has become the primary focus” (Miller, 2008, p. 398).

While this function of social media exchanges can be particularly valuable during a global pandemic and social isolation, it poses long-term problems related to online discussions and their potential to create new and valuable meanings for those involved. Instead of being exposed to a variety of points of view and learning from them, we rarely take the perspective of others, especially those different than us (Glǎveanu et al., 2018). Moreover, malevolent forms of creativity often emerge in online spaces, growing out of information bubbles or information silos and an “us versus them” mentality that both comes from and spills over into society and politics (de Saint Laurent et al., 2020). Both these consequences are starting to be documented in relation to COVID-19. We notice, for example, that “much of our public knowledge about the pandemic comes via advanced technology, through new media that has never before been tested during a disaster of this scope and size” (Wiederhold, 2020, p. 275) and that, in these circumstances, misinformation and conspiracy theories can flourish (Sharma et al., 2020; Tasnim et al., 2020). But there are also positive signs of “fighting back” through creative, social media activism (see Ferrara, 2020) in which people come together to promote positive social change and to build, collectively, fairer, more tolerant, and more reflective societies (Harrebye, 2016).

It is this complex context that offers the background for our research. What we are primarily interested in is not the nature or quality of the information circulating on social media about the pandemic. Instead, we focus on the creativity of coronavirus memes posted on a dedicated Reddit community. This allows us to raise questions about what makes a coronavirus meme creative and what kind of creative processes might be at play both in the construction and in the reception of such digital artifacts. We start the paper with a more general overview of creativity and social media in which we define Internet memes and consider their main properties. Then we turn toward a sociocultural theory of creativity that can shed light on online conversations about the pandemic. We apply this conceptual frame to the study of 480 memes, and, through a triangulation of quantitative and qualitative methods, we conclude about what matters most in appreciating online creativity and why some memes are seen as more creative than others. Implications for online creativity and our understanding of the pandemic are outlined at the end.

## Creativity, Internet Memes, and Social Media

A broad and important question is what the Internet and online participation are “doing” to our creativity. While this paper does not directly address this question (for a more detailed discussion, see Literat and Glǎveanu, 2016, 2018), it is undeniable that an increased number of people choose to express themselves creatively online. On the one hand, social media spaces offer new opportunities for collaboration, encountering different views, learning from others, and participating in a more open and democratic manner. On the other hand, there are still important barriers to creative participation in online space, most of all the tendency to look for similarity rather than difference and to build cohesive communities based on identity rather than debate. This tends to happen also in offline spaces, certainly, but social media chats, threads, and groups offer the right affordances for a quick and continuous exchange of messages and the uncritical circulation of “fake news.” And yet, the same media offer also their own “solutions,” supporting creative and pro-social activities like activism online and crowdsourcing (see Literat, 2012). The study of creativity on social media, in its benevolent and malevolent forms, is growing, leading to the recognition of online spaces as a domain of creativity in its own right (Kaufman et al., 2017).

Some of this research focuses on social media innovations and the way users both engage with them and drive them forward. Since such investigations are just beginning, Ratten (2017) formulated recently several propositions that need empirical testing, for instance that social media innovations are enhanced by team creativity, collective flow, and entrepreneurial skills and knowledge. And yet, it can be assumed that it is only a relatively small number of users who get to innovate online in terms of the technology itself. Most others engage in mundane forms of collaborative creativity and learning in social media environments. Peppler and Solomou (2011) documented this, for instance, in relation to a specific environment, *Quest Atlantis*, and the participation of 85 people designing virtual architecture. Their findings not only document processes of creative collaboration online but point equally to the social and cultural nature of their expression.

One of the most interesting contexts in which to study social media creativity, however, has to be that of the emergence, circulation, and transformation of memes.

“In the space of a decade, internet memes have gone from quirky, subcultural oddities to a ubiquitous, arguably foundational, and digital media practice. From Comedy Central’s television program Tosh.0 to the endless listicles of Buzzfeed, an entire media infrastructure has developed to report on, disseminate, and dissect the newest piece of digital culture to emerge, whether that is weekly, daily, or hourly. (…) Memes were important before they were ubiquitous because they represent a practice of vernacular creativity, a blending of folk practices (such as storytelling) with contemporary media savvy and skill” (Miltner, 2018, p. 412).

The notion of meme finds its origins in the work of evolutionary biologist Richard Dawkins who, drawing an analogy with genes, considered a meme to be a basic cultural replicator used to spread ideas and behaviors among individuals and within society (see Dawkins, 1989; also Blackmore, 2000). The ancient Greek word *mimema* stands for “that which is imitated” or for “imitated thing.” And, indeed, Dawkins saw memes—from wishing someone a happy birthday to fashion trends and more complex religious beliefs—as cultural forms that spread through imitation. In his words, “just as genes propagate themselves in the gene pool by leaping from body to body via sperms or eggs, so memes propagate themselves in the meme pool by leaping from brain to brain via a process which, in the broad sense, can be called imitation” (p. 192). While making some important contributions to our understanding of how some ideas and cultural practices spread far and wide, the meme theory has serious shortcomings. Key among them, and relevant for our discussion of Internet memes (which are a specific class of cultural units of meaning transmitted online as opposed to other cultural artifacts from the non-digital world), is the fact that memes are considered to replicate and transmit, almost out of their own “volition,” without much change. This contradicts online practices whereby these cultural products reflect human agency and constantly transform in the act of sharing.

Internet memes, called memes in this paper for the sake of simplification, have been defined as “remixed, iterated messages which are rapidly spread by members of participatory digital culture for the purpose of continuing a conversation” (Wiggins and Bowers, 2015, p. 1886). Or, in more simple terms, a meme is “a piece of culture, typically a joke, which gains influence through online transmission” (Davison, 2012, p. 122). The notions of *remixing* and *iterations* mark the fact that memes are, ultimately, outcomes of creativity that is, at once, individual and cultural; meanwhile, online transmission is an ecological marker of success and real-life “value.” In fact, memes serve a series of practical aims, from the pleasure of playing with language and images to displaying identity and fostering belonging (Carter, 2004). Following Zappavigna, Willmore and Hocking (2017) note and exemplify a series of key structural characteristics of memes like phrasal templates, catchphrases, image macros, and initialisms. In their words:

“Phrasal templates (also referred to as snowclones) involve formulaic sentence patterns with slots allowing content to be changed. A popular example is the ‘In Soviet Russia, [object] [plural verb] you!’ template. Catchphrases involve repeated whole phrases—for example, ‘Come at me bro’ or ‘Shut up and take my money.’ Initialisms involve the first letter from each word in a group or phrase. The most renowned of these is LOL (laugh out loud). Image macros—now one of the more common memetic forms on the web—involve an iconic static image overlaid with a humorous written text, typically using the Impact font” (p. 141).

There are several recent typologies developed in media studies that help researchers analyze memes. For example, Davison (2012) proposed three main components: manifestation, behavior, and ideal. The manifestation of a meme stands for its external, observable elements. It is the record of its existence, the arrangement of particles that bring it into being. In contrast, a meme’s behavior designates the actions performed by the individual in service of the meme (e.g., take a picture); these behaviors create the manifestation. Finally, the ideal of a meme is the concept or idea it represents. This dictates the behavior which, as we saw, generates the manifestation. So, for example, a behavior is to take or collect a funny cat photo which, once posted online, becomes a manifestation whose ideal (or meaning) might be that cats are funny. A similar typology has been proposed by Shifman (2013) who differentiates between content, form, and stance. Content is similar to ideal, form to manifestation and stance relates to tone and style of communication as well as the issue of who is entitled to participate and how. While these analytical categories do not guide our investigation in this study, it would be useful to apply them in the future to the case of coronavirus memes.

There have been, to date, few studies explicitly focused on the creativity of memes. A notable exception is the proposal by Willmore and Hocking (2017) to study memes using a framework for linguistic creativity. Their focus was to identify those markers of the creativity of language—e.g., repetition, rhetorical play, pattern reformulation, and punning—and the way they become manifest in memes. While this is highly suited for capturing the conversational nature of social media creativity, it does not account, for example, for the fact that memes cannot be reduced to text alone. What we are suggesting in this paper is to use a sociocultural framework, the perspectival model, in order to examine the creative processes involved in both the generation and reception of a meme. Moreover, there is no research yet, to our knowledge, that investigates creativity indicators (like originality, value, or elaboration) for this particular group of cultural and creative outcomes or examines how these indicators relate to each other. It is assumed, for instance, that “creative ideas are those that can be seen as spreadable” (Peppler and Solomou, 2011, p. 12) but what do people appreciate most about them?

Our research question is, thus, which sub-criteria of creativity—surprise (or the degree to which the meme is unexpected), meaningfulness (the degree to which they are understandable), humor (the degree to which they are funny), and elaboration (the degree to which they are carefully crafted)—best predict a meme’s creativity rating.

## Online Creative Processes and the Pandemic

As already mentioned in the previous section, Internet memes should represent a highly interesting field of study for creativity researchers, pointing them to the perennial issue of the relation between new and old, between similarity and difference in creative work. On the one hand, every meme is unique in one way or another, a product of the constant mixing and remixing of elements Wiggins and Bowers (2015) talked about. Even when they are a repost, memes can receive a different title or, by contrast with the other memes present on the thread, lead to new meanings, ideas, and discussions. Indeed, the creativity of a meme should not be reduced to its manifestation (according to Davison) or content (according to Shifman). While both contribute to identifying what has been created, the context of the meme, its uses, and consequences for wider communities of online users ultimately determine their success and level of creativity.

On the other hand, memes often use templates that are repetitive in nature and are firmly anchored in a specific meme tradition and popular culture. However, this should not decrease their creativity. As studies of other domains show, for example craft (see Glǎveanu, 2013a), repetition should not be understood as mindless replication (as in the meme theory initiated by Dawkins). On the contrary, Internet memes are defined by the productive *tension* between using existing cultural and digital “traditions” in order to add to them and to keep them alive and going. Even when similar themes and formats are being used, the messages conveyed and their contexts are changing and, therefore, their interpretations change too. Besides, there are a myriad of genres and sub-genres available:

“As Shifman (2013, p. 99) quips, ‘in theory, all Internet users are free spirits, individuals who take their unique path to the hall of digital fame. In practice, they tend to follow the same beaten tracks of meme creation.’ These ‘beaten tracks’ are genres, ‘socially recognized types of communicative action’ that are the ‘keys to understanding how to participate in the actions of a community’. There are dozens of genres of internet memes that have their own rules, structures, stylistic features, themes, topics, and intended audiences (Shifman, 2013). Some of the most recognizable meme genres include flash mobs, recut trailers, rage comics, lip dubs, image macros, and exploitables” (Miltner, 2018, p. 414).

And then there is the question of the author or creator. Traditionally, creativity researchers are accustomed to being quite clear as to who generated a given creative idea or object and keen to study his or her personality and cognitive dispositions. Memes most of the time eschew individual attribution and are embedded within wider systems and practices that make finding their “origin” even more difficult (Davison, 2012). They are, as such, a perfect example of *collective* forms of creativity and, for this reason, tend to fly below the radar of mainstream creativity researchers. In addition, there is often a cultural assumption, at least in the West, that creative outcomes are valuable, and their value needs to be credited to their creator (the whole premise behind copyright laws). Since memes are inexpensive to produce, and are produced by a large number of people, they are easily seen as requiring less talent and a lower level of investment. It is easy thus to disqualify memes as worthless or too mundane and not recognize the social, little-c creativity clearly invested in their making (Craft, 2001; Kaufman and Beghetto, 2009).

Memes have been recognized as *cultural artifacts* for a long time. Wiggins and Bowers (2015) offer, for instance, three reasons why memes should be seen as such: their virtual physicality, social and cultural connection, and purposeful production and consumption. What we need is to also appreciate them as *creative artifacts* (Glǎveanu, 2013b), using cultural resources in order to generate new and meaningful cultural resources. This aligns well with the sociocultural approach to creativity (see Glǎveanu et al., 2019), an approach that starts from the premise that all creative outcomes are, ultimately, cocreated within the interaction between individuals, groups, and cultures. This dynamic is perfectly illustrated by the creation, circulation, and transformation of memes which offer sociocultural researchers a paradigmatic case of creative expression.

The perspectival model (Glǎveanu, 2015) assumes that creativity emerges out of acts of repositioning, perspective-taking, and dialogues of perspectives. Rooted in pragmatist accounts of action and experience (in particular Meadean and neo-Meadean scholarship; see Gillespie and Martin, 2014), it considers that we are all rooted in various physical, social, and cultural–symbolic positions within our environment and that it is by changing this position, and especially exchanging it with that of others, that we get to develop new perspectives or understandings of the world. Finally, it is by placing old and new perspectives in dialogue with each other that new and meaningful ideas emerge—this is, according to the perspectival model, the nature of a creative process.

When we consider memes and their creation and distribution, it is easy to see how they reflect *multiple perspectives* on their topic. These tend to be captured by the difference between different images, different pieces of text, or the relation between text and image. Memes also construct specific viewer positions from which they become comprehensible by using linguistic means like “when you…,” or playing with the notions of “me” versus “everybody.” The cultural, interactive nature of social media environments have been repeatedly noted before (Peppler and Solomou, 2011), but what different authors start advancing is the *conversational* nature of the meme genre. According to Willmore and Hocking, “whereas many social media platforms—for example, chat rooms, instant messaging, or comments sections in blogs—are specifically organized to facilitate a way of interacting that resembles spoken conversation, Internet memes are perhaps less identifiable as a communicative genre” (2017, p. 144). This is very much in line with the dialogical premise of the perspectival model and encourages us further to study the creativity of memes as a dialogue or conversation between different points of view.

What might be the “outcomes” of such dialogues? We propose here that the creative processes involved in generating and interpreting memes related to the pandemic—and, presumably, outside of this context as well—can be described in terms of (a) *familiarizing* the new; (b) *de-familiarizing* the old; and (c) *re-familiarizing* the old with the help of the new. To take these in turn and specify operational ways of differentiating between them, the first dynamic advances a familiar perspective on an unfamiliar topic or social object (like the virus or the unprecedented measures to address it are in our case) with the aim of making it more understandable. For instance, comparing the pandemic with the plague familiarizes it for us within the register of historical events and great, frightening diseases. But the pandemic gives us the occasion to also deconstruct objects or habits we are accustomed with. For example, the joy of spending some time at home, relaxing, isolated from the craze of the outside world, is put in a new light by imposed isolation. What was once assumed as commonplace and known is destabilized by a new and unexpected, even opposing perspective. Last but not least, there are situations in which the old and the new perspective are held in balance with each other and, in doing so, add meaning to a social object that does not deny or exclude old understandings. For instance, if considering staying at home as a punishment negates the relaxation of this same act in the past, seeing a “health worker” as a “hero” adds a new level of meaning to this category of people without replacing existing perspectives.

Our second research question, explored qualitatively, is how familiarization, de-familiarization, and re-familiarization are used in memes that score highly on creativity and its subcomponents (surprise, meaningfulness, humor, and elaboration). We do not assume any hierarchy between these processes when it comes to overall creativity because, in the end, they are all based on dialogues of perspective and invite such dialogues in viewers.

## Methodology

The study reported in this article used a triangulation of quantitative and qualitative methods to answer the two research questions mentioned above:

1. Which of the sub-criteria of creativity—surprise, meaningfulness, humor, and elaboration—best predict the overall creativity scores of coronavirus memes?
2. How do highly surprising, meaningful, humorous, elaborate, and creative coronavirus memes use the processes of familiarization, de-familiarization, and re-familiarization?

Data were collected from a dedicated Reddit community (r/CoronavirusMemes). A total of 22,000 memes, posted between 21st of January until 17th of May, have been downloaded. From these, 480 memes were randomly selected from 12 groups of memes (based on 3 time periods × 4 levels of success on Reddit or upvotes score^1^) and assigned to 8 samples of 60 memes each. These were rated by three coders each for five criteria on a 5-points Likert scale within an online questionnaire: surprising (how unexpected the meme is), meaningful (how easy it is to understand its meaning), funny (how humorous the meme is), elaborate (how well made the meme is), and creative (coders were asked to use their own definitions of creativity in this regard, in agreement with the Consensual Assessment Technique; see Amabile, 1982). Surprise was preferred to novelty or originality given that memes are, by definition, supposed to remix cultural material; as such, surprise as a personal, emotional reaction of the coder is more easily detectable than the other two. Meaningfulness was also preferred to value, task appropriateness or suitability since a meme is appropriate when its meaning can be easily grasped and, thus, can be more readily transmitted. Elaboration, a less used criterion for scoring divergent thinking tests, is included here given that memes can vary in relation to how much attention to detail and mastery the (largely anonymous) authors demonstrated in making them. Finally, humor was added given that memes are often defined as funny, even if this is not always the case. The coders were presented, for each meme, with only the picture and title given on the reddit post where the picture had been submitted (not the karma score or other details).

The questionnaire ended with questions about basic demographics (age, sex, and nationality) and, in addition, level of proficiency in English, knowledge of memes and meme culture, frequency of using Reddit, and frequency of visiting and/or submitting to the r/CoronavirusMemes. Coders were recruited from Prolific based on their use of Reddit and they were compensated for their time (3.75 GBP for 30 min). The platform Prolific was chosen—instead of the more popular Amazon TURKS—because its organization, rules, and levels of compensations and rules are compatible with the ethical standards of academic research (e.g., participants are not penalized if they decide to leave the study; Palan and Schitter, 2018). The study abided by the ethical guidelines of the APA, respondents were informed about their rights, and they could terminate their participation at any time.

The coders were a majority of men (70.83%) and generally young (mean = 30.29; std = 10.16), in line with the demographics of reddit users. They declared being, on average, moderately to extremely knowledgeable about memes (91.67%) and using reddit at least once a week (83.33%). None of them were familiar with the specific subreddit where the memes were collected, which is not surprising considering that reddit counts more than 2 million communities and r/CoronavirusMemes counted 83.786 subscribers on May 18th—when the final data was collected—out of more than 220M users.

Given that each image was rated by 3 coders on 5 criteria for a total of 480 images, our quantitative dataset ended up including 7200 unique ratings in total (or 1440 ratings if grouped by criteria). In total, 24 users coded the memes, with each coding one of the 8 samples of 60 images. While our main interest laid in the correlations between the different ratings given by our participants, we also looked at intercoder reliability to estimate how convergent the participants were in their understandings of what constitutes a creative, meaningful, elaborate, surprising, or funny meme.

In order to examine the relation between each criterion, we analyzed the ratings in two manners: first, we used a mixed-effect model to analyze how much of the creativity scores given by each participant could be predicted by the four other criteria. However, because of the limited number of participants in our study, these results have an exploratory value. Second, we used a repeated measure of correlations, analyzed in R, in order to look at how the ratings given by the participants on each criterion related to each other, an analysis more fitted to the size of our dataset. Both methods allowed us to account for the fact that scores given by the 24 coders on each of the 60 memes they were presented with are, in fact, repeated measures. A total of 10 memes were afterward selected for qualitative analysis (from the higher ranking on the 5 criteria scored, eliminating repetitions, based on averaged ratings by the 3 coders), and two more memes that obtained the lowest creativity scores were included for contrast. These 12 memes were subjected to a perspectival analysis that considered (a) what perspectives were present in the image and (b) how these perspectives, new and old, related to each other. In particular, we focused on the processes of familiarization, de-familiarization, and re-familiarization outlined above and, thus, on the nature of the perspectival dialogues established within the meme.

## Results

### Quantitative Findings: Creativity Ratings

On average, the coders rated the different criteria slightly below the mean, with the exception of meaningfulness (see Table 1). This is also an encouraging sign that most memes “made sense” to the three evaluators.

**TABLE 1:** Descriptive statistics for creativity related ratings.

	Mean	Std.	Median
Creative	1.74	1.36	2
Surprising	1.75	1.49	2
Meaningful	2.11	1.41	2
Elaborate	1.69	1.32	2
Funny	1.62	1.44	1

#### Inter-Rater Reliability

While it was not our primary aim to obtain a consensus agreement of the participants over what constituted or not a creative meme, we were curious about the potential convergence of the ratings attributed to the memes. With this purpose in mind, we estimated the inter-rater reliability (IRR) for each sample—so for each unique group of three coders—and each criteria, using Krippendorff’s alpha, considered one of the most reliable IRR measures (Hayes and Krippendorff, 2007). To account for the fact that different coders might be more or less lenient in their evaluation, the IRRs were calculated both on raw and on normalized scores. While the latter—see Table 2—yielded slightly higher scores, it is clear that the IRRs are beyond suboptimal. Indeed, convergence is considered to be achieved with a Krippendorff’s alpha above 0.66, and negative scores—as we obtained in multiple cases here—are extremely rare. While the size of our sample makes it impossible to reach any firm conclusion, the scores do suggest that the issue does not primarily lie with the sample size but with the subjective understanding of the categories by the participants. This is because the IRR scores we obtained are not simply low but indicate that the coders were divergent on their ratings (Hayes and Krippendorff, 2007), although no lower threshold has been proposed to confirm divergence—the aim being generally to reach convergence.

**TABLE 2:** Inter-raters reliability (Krippendorf’s alphas).

Criteria	Meaningful	Surprising	Creative	Elaborate	Funny
Sample 1	0.19	−0.07	0.05	0.05	0.02
Sample 2	−0.06	0.02	0.06	−0.02	−0.08
Sample 3	0.01	0.05	0.03	0.05	0.11
Sample 4	0.02	0.02	0.12	0.02	0.27
Sample 5	−0.03	−0.05	0.06	0.14	0.06
Sample 6	0.14	0.05	0.07	0.03	0.19
Sample 7	0.26	0.38	0.22	0.11	0.28
Sample 8	0.01	−0.11	0.15	−0.08	−0.04

#### Mixed-Effect Modeling

We used mixed-effect modeling to estimate how much of the creativity scores given by the participants could be predicted by their ratings of the other 4 criteria. Indeed, mixed-effect modeling allows for the analysis of hierarchical data that is grouped in clusters of dependant observations. In our case, individual observations—each group of five ratings given by one coder on one image—are grouped at two levels: first, at the level of the coders, whose ratings on different images are not independent from each other, and second, at the level of the sample, given that each coder encountered one of eight samples of images and that these samples may diverge in ways we could not control. The models were fitted using the lmerTest package in R (Kuznetsova et al., 2017) and the R squares calculated using the MuMIn (Multi-Model Inference) package and following the recommendations of Nakagawa and Schielzeth (2013) for calculating R squares on mixed-effect models. The confidence intervals for the explained variances were calculated using Maindonald and Braun’s (2006) method and the lme4 package (Bates et al., 2015).

Three models were fitted in turn, all with creativity as the dependant variable. The first model included the four criteria rated—meaningfulness, surprise, elaboration, and humor—and a random effect for the coders and samples. In other words, the model calculated a fixed effect of each criterion and added a varying intercept for each coder and each sample. The second model added the criteria to the random effects of the coders: that is, the slope of the effect of each criterion was allowed to vary for each coder. The third model added, as a fixed effect, two characteristics of the coder and their interaction: Reddit usage and familiarity with memes. For simplicity, we show here the R squares and the comparison of the models (using an ANOVA), and the full results only for model 2, that proved to be the best fit based on the AIC (with delta > 10). The comparison of the three models is presented in Table 3 and model 2 in Table 4.

**TABLE 3:** Mixed-effect models with the creativity ratings as the dependent variable.

	*R*^2^ fixed effects	*R*^2^ fixed + random effects	Df	AIC	BIC	Log likelihood	Deviance	Chi^2^	Chi Df	*p*-value > Chi^2^
Model 1	0.731	0.752	8	2829.4	2871.1	−1406.7	2813.4			
Model 2	0.731	0.766	22	2802.5	2917.2	−1379.2	2758.5	54.881	14	<0.001***
Model 3	0.724	0.760	25	2807.5	2937.8	−1378.7	2757.5	1.015	3	0.798

Model 1: Creative ∼ 1 + Elaborate + Funny + Surprising + Meaningful + (1 | coder) + (1 | Sample). Model 2: Creative ∼ 1 + Elaborate + Funny + Surprising + Meaningful + (1 + Elaborate + Funny + Surprising + Meaningful | coder) + (1 | Sample). Model 3: Creative ∼ 1 + Elaborate + Funny + Surprising + Meaningful + meme_culture + reddit_use + meme_culture * reddit_use + (1 + Elaborate + Funny + Surprising + Meaningful | coder) + (1 | Sample). ^∗∗∗^ Significant at less than 0.001.

**TABLE 4:** Results for model 2.

Fixed effects:									
Variable	Estimate	Std. error	df	*t*-value	*p*-value	Correlations^1^			
(Intercept)	0.120	0.063	10.470	1.888	0.087				
Elaborate	0.518	0.034	12.623	15.221	<0.001***	0.066			
Funny	0.259	0.039	18.715	6.657	<0.001***	0.073	−0.650		
Surprising	0.118	0.029	13.035	4.082	0.001**	−0.598	−0.128	−0.334	
Meaningful	0.061	0.025	24.624	2.395	0.025*	−0.228	−0.002	−0.430	−0.054

**Random effects:**							

**Groups**	**Name**	**Variance**	**Std. deviation**	**Correlations^1^**			

Coder	(Intercept)	0.050	0.223				
	Elaborate	0.011	0.105	0.27			
	Funny	0.022	0.150	0.14	−0.91		
	Surprising	0.009	0.096	−0.95	0.01	−0.39	
	Meaningful	0.004	0.063	−0.03	0.61	−0.70	0.07
Sample	(Intercept)	<0.001	<0.001				
Residual		0.425	0.652				

*^1^The correlations are between the estimates of the slopes and the intercepts, not between the variables themselves. ^∗∗∗^ Significant at less than 0.001.*

This analysis shows that the 4 criteria included capture a significant part of what the participants thought would constitute a creative meme: model 2 explains 76.6% of the variations in creativity scores, with 18.50% of the variance imputable to the random coder effects. The correlations between the different random effect estimates indicate positive relations between elaboration, surprise, and meaningfulness (the more one influences the creativity rating, the more the others do too) and negative ones between humor and the other criteria (the more humor correlates with the creativity rating, the less the other criteria do too). In other words, it seems that some participants judged the creativity of a meme to be mainly the product of how funny it was, while others gave more importance to the elaboration, meaningfulness, and uniqueness of the meme.

#### Repeated-Measure Correlations

**FIGURE 1:**
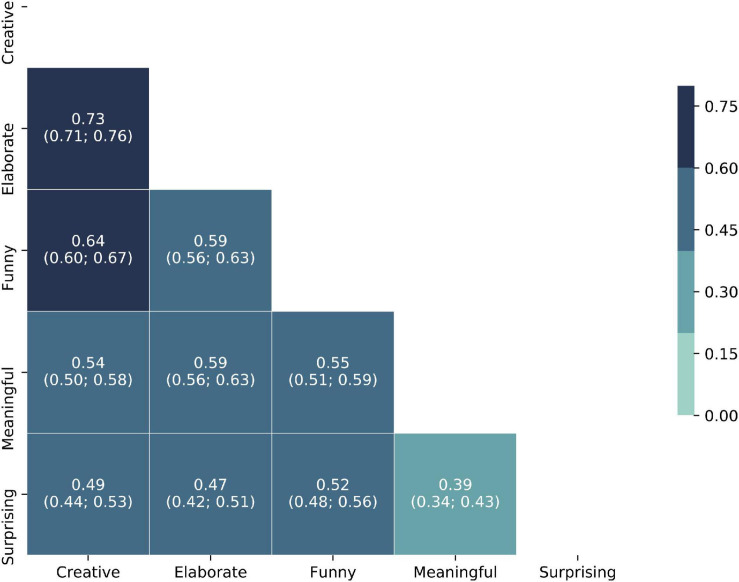
Heat map of the correlations between the criteria (confidence interval: 95%, all values significant at *p* < 0.001).

In order to explore further the relations between the different criteria, we analyzed their correlations, while taking into account the interdependence of the measures, given that each coder produced 60 groups of ratings. We thus used the method proposed by Bakdash and Marusich (2017) to evaluate repeated-measure correlations, and the associated R package rmcorr, which also calculates confidence intervals. The results are presented in Figure 1. As can be seen in the figure, the creativity rating correlates the most with elaboration, then humor, and finally meaningfulness and surprise. It thus seems that how well a meme is crafted mattered more, for our participants, than how funny they found it, a surprising result given how humor is seen as central to meme culture. It is also worth noting that while all the criteria are quite strongly positively correlated, meaningfulness and surprise share the smallest overlap, indicating that it may be difficult for memes to both make sense for a wide audience and yet be novel. In order to explore these different findings further, a qualitative analysis was then carried out.

### Qualitative Findings: The Creative Process

A difficult question to answer has to do with the creative processes involved in the creation of memes. This is because, as mentioned before, meme creators are largely anonymous and, even if we were to identify the maker of a specific meme, the latter is arguably more reflective of a collective Internet culture than the individuality of the author. This second observation makes the study of meme interpretation—and any creative processes involved in this act of meaning-making—difficult given that Internet culture is multifaceted and one and the same meme will gain different significations and be received differently by various users. In this research, we chose to ask users to quantitatively rate memes for surprise, meaningfulness, humor, elaboration, and creativity, but not to describe or discuss the meme itself (mainly for pragmatic reasons, as the latter would have been more time consuming). It is to be noted that some of the memes included below, and considered as creative by our coders and, in some cases, by the Internet community, might be less sensitive in their depiction of certain groups (e.g., Asian people) and therefore their wider appreciation could vary also because of this.

And yet, what we will attempt here is to interpret the processes involved, potentially, in *both* the creation and reception of memes based on the perspectival model of creativity briefly described in the section “Online creative processes and the pandemic.” We are confident we can do this for two reasons. First, the perspectival approach is general enough to be applicable to a wide range of creative artifacts, including memes. Its assumption that a meme reflects various perspectives on a given topic, new and old, is reasonable and in line with existing literature on memes as a communicative genre (Willmore and Hocking, 2017; Miltner, 2018). Second, this approach is suitable for analyzing the creation and reception of memes given that these are both *meaning-making processes* (like familiarization or de/re-familiarization) and, as such, driven by the perspectives people discover in or attribute to a creative artifact. Having said this, we do not assume that the findings that follow reflect the experience of each and every meme creator and user but, as is the case with qualitative research, illustrate some essential mechanisms that can be explored further in qualitative and quantitative studies.

We have chosen a total of 12 memes for this study given that our aim is not to generalize to the entire set of memes but to exemplify basic creative processes that can be used to examine coronavirus memes and not only. These were selected based on averaged scores from the coders and represent the top memes for surprise, meaning, humor, elaboration, and creativity (excluding duplicates), alongside the two least creative memes.

**FIGURE 2:**
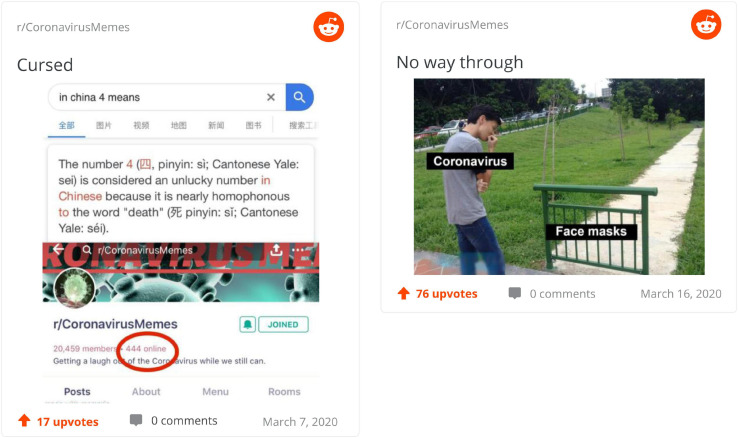
Top creative memes.

Figure 2 depicts two of the memes scored most highly for creativity in the sample. On the left side, on top we have information about the number 4, considered to be unlucky and associated with death in Chinese and, at the bottom, we are made to notice the presence of “444 online” in the coronavirus memes Reddit tread. There are thus two perspectives presented on the number 4 which, for most of us, is a number like any other while for some people, culturally, it stands for something completely different. Then, we have an implication that bad luck or death is marking now the Reddit community posting memes. Whether this association is found to be funny or “informative” (alluding to conspiracy theories) is secondary. What we can notice is the *re-familiarization* of number 4 which stands to represent a total (like 444) and, presumably, a warning. On the right side, the meme offers an allegorical representation of coronavirus as a man looking at a fence, here standing for a facemask. The humorous aspect of the meme comes from the ease with which the man could bypass the fence, an allusion to the ease with which the virus escapes our protective equipment. The perspectives involved in the meme play on this duality between man–fence virus–mask, brought together by the same idea: that it is easy for one (man/virus) to overcome the other (fence/mask). In the terms we use here, this is an example of *familiarization* of something previously unknown and unfamiliar—the coronavirus—by anchoring it and the measures taken against it within a register we are highly familiar with—the everyday encounter between the man and a tiny obstacle.

**FIGURE 3:**
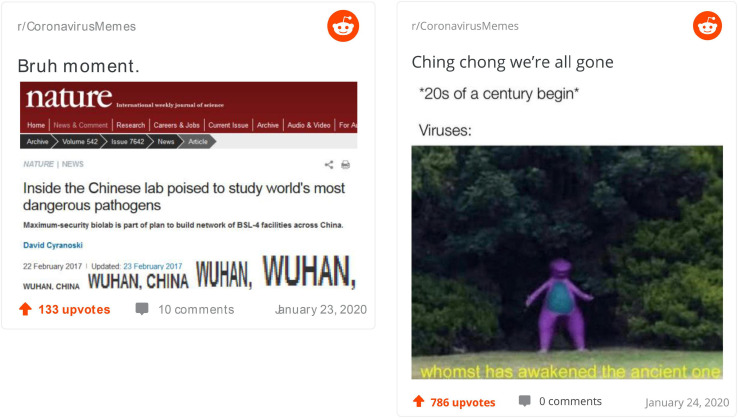
Least creative memes.

It is interesting to ask if the same processes of creative meaning-making are as easily displayed by memes appreciated as least creative by coders. Two examples of such memes are offered in Figure 3. On the left, we notice an article seemingly from Nature (although obviously tempered with) about a Chinese lab aiming to study the world’s most dangerous pathogens. Instead of the text of this article we find written increasingly large “Wuhan, China, Wuhan, China, Wuhan, Wuhan.” The perspective we are offered is that the virus is manmade in a lab in Wuhan, China. This is meant to be in conflict with a more common assumption about the natural origin of the virus. The meme tries to *familiarize* the coronavirus as an engineered pathogen, a type of misinformation whose creativity has been rejected by our coders. On the right, we see a man dressed up in a dinosaur-like costume coming out of the woods (presumably an ancient monster) and, at the top, a suggestion that a major virus comes out every second decade of each century (again a misinformation). This is not a new perspective or information in any way and what it tries, at best, is to have a comedic effect by *familiarizing* the virus as an old demon that has been reawakened. While we note that both these memes reflect versions of familiarization, this does not imply that this process is less creative than de- or re-familiarization. As we saw just above, familiarization can also underpin some of the most creative memes. It is the content (misinformation) and the fact that the template for both has been overused that made these particular attempts at familiarization less successful.

**FIGURE 4:**
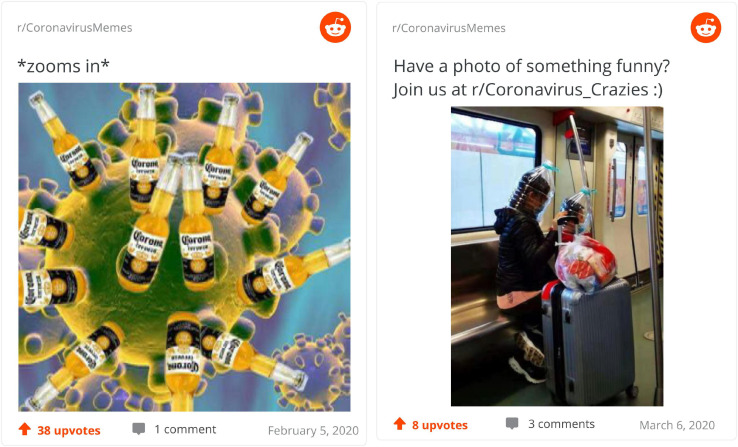
Top surprising memes.

Figure 4 depicted two of the memes considered to be most surprising. The meme on the left is simple yet effective in its design, replacing the small spikes of coronaviruses with Corona beers. This is a good example of *re-familiarization* of the classical image of the virus that we see online based on the association, through the name, with the beer. An implied perspective might be that, at a closer look, Corona beer does have something to do with the pandemic, an unexpected conclusion to reach after all (but not entering the realm of misinformation as the image is clearly fabricated). On the right, we are faced with another surprising image, this time presumably real rather than altered. Two people travel on the metro with protective equipment made of huge plastic bottles that cover their whole head. This image *de-familiarizes* for us both plastic bottles and facemasks, adding the new and unexpected perspective that the former can serve as the later.

**FIGURE 5:**
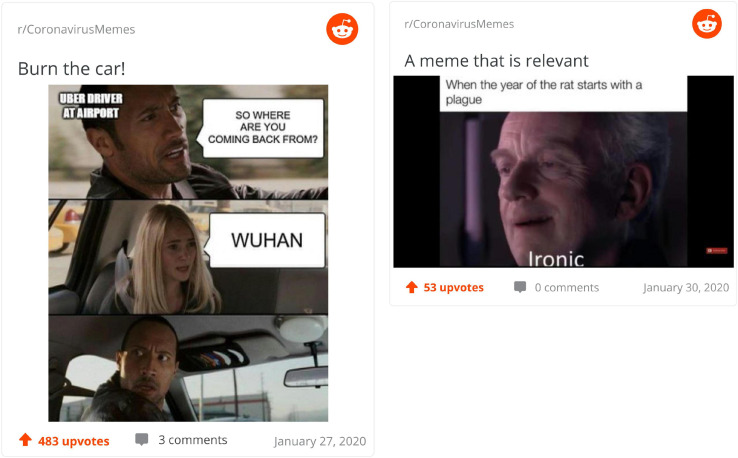
Top meaningful memes.

In Figure 5, we find two of the top memes scored for meaningfulness or the ease with which they can be understood. On the left, a common meme template is used to capture the presumed conversation between an Uber driver picking up people from the airport and his passenger. The comedic effect is ensured by the face of the driver upon hearing that the girl in the back seat is coming back from Wuhan. Two perspectives are contrasted here: the normal curiosity of a driver trying to make small talk is contraposed with the seriousness of the topic (the girl’s point of departure). The meme *de-familiarizes* for us the act of driving and talking to someone from mundane to potentially dangerous in a way that is immediate to grasp. On the right, the meme is anchored in the Star Wars universe, where the emperor calls ironic the fact that the year of the rat started with a plague. The perspective associated here concern the Chinese zodiac, on the one hand, and the fact that rats are not only astrological signs but disease-carrying pests. This analogy is anchored as well in history (through the reference to the plague). It is an example of *re-familiarization* of the rat as, as the same time, a zodiac sign and as an animal historically related to the plague, two interpretations consequential for the current crisis.

**FIGURE 6:**
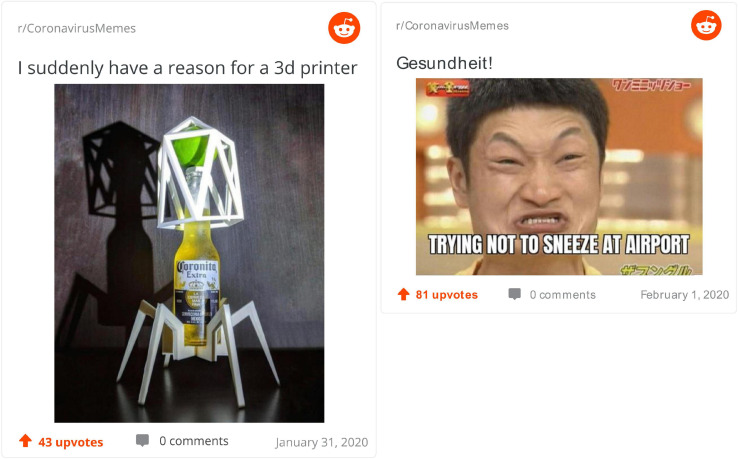
Top funny memes.

Figure 6 shows two memes scored highly on humor by their three coders. On the left, we see a Corona beer placed on designed structures that make it look menacing (including through the shadow it casts on the wall behind) and virus-like. Once more, we are presented with an analogy between the well-known beer and virus, based on their common name. But, instead of re-familiarizing the classic image of the virus, we more *de-familiarize* how we normally see Corona beers. The perspective of having a good time drinking beer is replaced here, in a humorous manner, by that of a dangerous thing to touch, little less drink. On the right, we find the squinting face of an Asian man “trying not to sneeze at the airport.” This alludes to the fact that we all, and especially people coming from Asia, avoid displaying in public symptoms of the virus. Through the airport, we have an added assumption that people who travel when sick are even more dangerous. The humor in this meme is mostly physical, through the face of the man (and it does seem to have some racist undertones), but it is also based on *de-familiarizing* the very mundane experience of sneezing—passing from the perspective of being largely inconsequential to that of highly suspicious.

**FIGURE 7:**
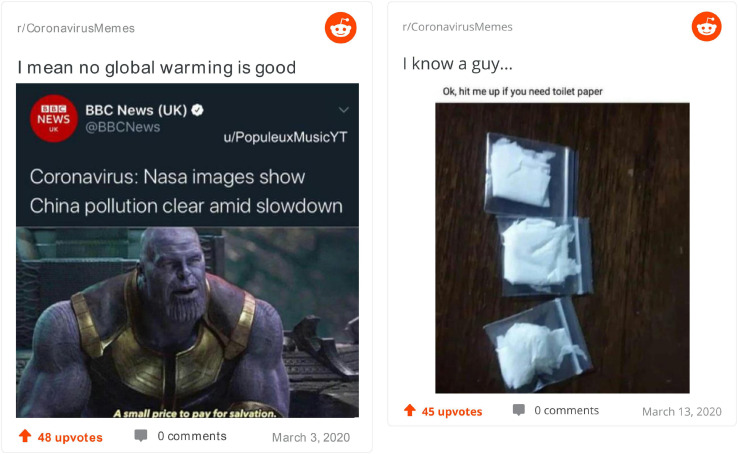
Top elaborate memes.

Last but not least, in Figure 7 we have two of the most highly scored memes for elaboration. On the left, the image of Thanos, from the Marvel Universe, is placed against the background of BBC News announcing that the pollution over China has been reduced amid the slowdown in economic activity. Thanos’ line that it is “a small price to pay for salvation” comes from his obsession, in the comics, with reducing the population in order to save the world. These perspectives about the environment, one coming from presumably factual information about pollution and the other from a fictional character and world, are strangely convergent. This *re-familiarizes* for us the notions of climate change, pollution and its reduction as both desirable and coming at the price of great sacrifices (despite what Thanos claims). It is an elaborate meme in the sense of bringing together well, in an artistic manner, two unconnected universes (real news and fantasy antiheroes). On the right, pieces of toilet paper in plastic bags gain the status of prized possessions (largely because they are sold in such small quantities). More than this, the image alludes to selling drugs, an interpretation accentuated by the text up saying, as a drug dealer would, “hit me up when you need toilet paper.” Two perspectives on toilet paper—as cheap and precious—are placed in relation to each other, *re-familiarizing* us with this mundane object. From a harmless, unimportant possession, toilet paper gains the status of prized possession that has an addictive quality for all those who are missing it. The high score on elaboration for both these memes might also come from the fact that their creators clearly took time to make them (e.g., to actually prepare the small bags).

## Discussion

A study of coronavirus memes is not only very timely but, as we hope to have demonstrated in this paper, it can shed new light on both creativity and Internet culture. Memes are widely considered to “act like a funhouse mirror for culture and society, reflecting and refracting the anxieties and preoccupations of a variety of social groups across a series of national contexts” (Miltner, 2018, pp. 412–413). And it is especially pandemic-related anxieties that they capture and help us make sense of in a creative manner. As mundane, little creative artifacts coming out of a highly participatory digital culture, memes also capture something essential about the processes of *distributed creativity* (Glǎveanu, 2014), namely, the fact that cultural content is constantly being recreated in and through processes of transmission. In this regard, Internet memes reflect less Dawkins’ conception of meme replication (1989) and are more aligned with social representations perspectives (see Moscovici, 1984) on the transformation of social knowledge in the process of circulating among various communities. Considering how intimately these processes are related to creativity, it is surprising that we do not have more research on meme creativity to date. While online culture has been of interest for a while (e.g., Greenhow et al., 2011), memes largely flew under the radar, perhaps also given the difficulties associated with analyzing image rather than text (Highfield and Leaver, 2016; Literat and Kligler-Vilenchik, 2019). As such, our study marks what we hope is the beginning of a new concern for digital and visual content in creativity research.

In this discussion, we will be guided by our two research questions and focus on the broader implications of our findings. Before answering them, however, it is interesting to note how low the correlations between our coders’ ratings were to begin with. This raises a challenge for consensual assessment (Amabile, 1982) as we suspect it has less to do with the fact that our coders were inexperienced (in fact, they were frequent Reddit users) and more with the specificity of the domain in question. Memes might be more difficult to score convergently than other cultural artifacts given their abundance online and deep anchorage within specific Internet subcultures (Davison, 2012). As such, what seems creative or funny or surprising for one evaluator might not look the same for another. We managed to circumvent this obstacle by analyzing the consistency of rating in the case of each assessor rather than averaging their scores for a particular meme.

When it comes to the relation between sub-criteria like surprise, meaningfulness, humor, and elaboration on the hand and creativity scores on the other, an unexpected result came about. The standard understanding of creativity, at least in psychology, pointed for decades to the importance of novelty/originality and value/appropriateness (see Stein, 1953). In terms of our research, these would translate into the criteria of surprise and meaningfulness. However, it was *elaboration* that correlated the highest with creativity, then humor and only finally meaningfulness and surprise. This raises very interesting questions for creativity researchers. Elaboration, operationalized in this study as attention to detail and nice execution of the meme, is inspired by the same criterion used in coding divergent thinking answers (in those cases, dealing primarily with material objects or linguistic outcomes, elaboration refers to how complete or detailed a product is). The “mastery” demonstrated in creating a meme and its appreciation are not unique to the domain of online creativity. In fact, they are also common in craft and folk art where a decorated egg, for example, is not judged as creative based on its novelty but its masterful renewal of an existing tradition (Glǎveanu, 2013a). It might seem farfetched to associate Easter eggs and other outcomes of traditional craft with the latest manifestations of technological advancement in digital spaces. And yet, the notions of *tradition and culture* are pertinent to both and elaboration seems to become important whenever a creative practice is largely based on mixing and remixing cultural content.

This is reflected as well in our qualitative analysis aiming to examine creative processes. The idea of familiarization and its variations refers back to the relation between the *new* and the *old* in meme culture. New perspectives might be applied to familiar objects or well-known perspectives used to make sense of strange and unfamiliar realities, like the coronavirus. But, in both cases, mastery is demonstrated in the smooth articulation between the expected and the unexpected, the familiar and the unfamiliar. Our qualitative study of highly performing memes illustrated the interplay, in online creative expression, between familiarization, de-familiarization, and re-familiarization. It was not our aim to claim one of the three processes above as more “creative” than the others. Given that, according to the perspectival model (see Glǎveanu, 2015), creativity is born out of a dialogue of perspectives, these dialogues can equally lead to a new perspective on what is (de-familiarization), an “old” view on what is new (familiarization), or the coexistence of old and new understandings (re-familiarization).

What matters most here is to understand the *tension* between perspectives and the insights and meanings emerging out of their juxtaposition and, sometimes, their clashes. For example, the mundane act of sneezing gains completely new (and alarming) significations during the pandemic (primarily, unfortunately, for people who look Asian). Or trivial pieces of toilet paper can gain a new status as valuable (and addictive) merchandise due to shortages and stockpiling. There is often a perceivable tension between realism and silliness, as observed in the Corona beer meme from Figure 4, in which the imagine is clearly fabricated and yet the beer bottles replace perfectly the virus spikes. The other meme in the same Figure, from public transport, is exaggerated and yet strangely realistic. Even the use of Thanos in the meme from Figure 7 looks silly when juxtaposed with the news but, at the same time, it makes sense for news about pollution (given the character’s story). Humor emerges from this kind of tensions and especially the unexpected association between contrasting perspectives (Chapman and Foot, 1976). This is interesting on two accounts. First of all, memes are supposed to be funny by definition (Davison, 2012), which should make them even more relevant for creativity researchers, at least those working within the sociocultural tradition. Second, being funny scored highly in relation to overall creativity in our study, second only to elaboration. This suggests that, on the one hand, humor should be theorized further in relation to online cultures and, on the other, that it should receive more attention in the psychology of creativity. In particular in view of the current pandemic, humor can very well be a coping mechanism (Kuiper et al., 1993), in addition to a meaning-making and community-building device.

There are a series of limitations associated with this study. First of all, low IRR might be due to the fact that we used novice raters in this research and, in these cases, it is usually recommended to have more than three raters (Kaufman et al., 2013). However, it is also to be noted that it is difficult to identify Internet meme experts as this is not an established domain but a relatively new and emerging one. Moreover, our interest was not to converge on a “true” creativity score for each meme but to study the way in which different ratings related to each other for one and the same rater. Connected to this, there is the issue of *interpretation*. As Miltner (2018) noted, following existing literature, memes “are successful because they allow different audiences to make their own meanings from the same media artifact; the specific element within each internet meme that strikes a chord will differ from person to person” (p. 414). This poses a challenge for scoring creativity and its dimensions and raises the issue of having a group of coders who are familiar with similar Internet subcultures. On the other hand, however, the fact that different readers will interpret [memes] and put them to use in varying ways” (Miltner, 2018, p. 414) is a reality taken into account by the sociocultural approach used in this study. From this perspective, it is not the consensus but rather *divergence* of views that should become the object of research (Glǎveanu, 2012). Following this approach through, though, would require going further in our analysis of memes and examining also how people respond to a meme online and the chains of conversation they initiate. As previous research in online environments has shown, “creative artifacts served as the foundation for dialogues that enabled communities at large to determine and negotiate their cultural values” (Peppler and Solomou, 2011, p. 12). As such, it would be useful in future studies to analyze both memes and the conversations they generate (see, for instance, Glǎveanu et al., 2018), ideally in more than one forum or social media platform.

## Conclusion

The study of memes, in this case memes dedicated to the coronavirus, as creative artifacts opens up new venues of research for both media scholars and creativity researchers. It can help the former by suggesting theories (like the perspectival model of creativity) and analytical tools (like the typology of familiarization, de-familiarization, and re-familiarization) that can be useful for exploring collaborative creativity in online and digital spaces. For the latter, it offers a new and rich domain of research—memes and social media—that can lead to new and surprising conclusions, for instance, an invitation to reconsider the role of mastery, elaboration, and humor in creativity within and beyond social media. Last but not least, this study hopes to shed new light on the current pandemic and Internet users’ reactions it. The creation and circulation of memes might seem like a minor pastime, but, under conditions of lockdown and social isolation, it might be a key component in what makes the difference between psychological and social adjustment and maladjustment. Further research on the role of creativity in relation to *meaning-making*, *well-being*, and *mental health* (Kaufman, 2018; Richards, 2018) is called for during and in the aftermath of the COVID-19 pandemic.

## Data Availability Statement

The raw data supporting the conclusions of this article will be made available by the authors, upon request.

## Ethics Statement

Ethical review and approval was not required for the study on human participants in accordance with the local legislation and institutional requirements. The patients/participants were not required to provide their written informed consent to participate in this study, in accordance with the local legislation and institutional requirements.

## Author Contributions

VG drafted the manuscript and was in charge of the qualitative analysis. CS was in charge of the quantitative analysis. Both authors contributed to the article and approved the submitted version.

## Conflict of Interest

The authors declare that the research was conducted in the absence of any commercial or financial relationships that could be construed as a potential conflict of interest.

## Footnotes

1 The time periods correspond to three different phases of the life of the subreddit r/CoronavirusMemes: (1) January 21st to March 8th, from the start of the community to its expansion into a large subreddit; (2) March 9th to March 29th, stage during which the number of daily submissions to the community was consistently high; and (3) March 29th to May 17th, period during which the level of activity on the subreddit contracted back to about 1/6th of its peak levels. The levels of success correspond to the number of upvotes received by a meme, relative to how many votes were cast that week (to adjust for the fluctuating size of the community): (1) bottom 50% of memes; (2) between 50 and 75%; (3) between 75 and 90%; and (4) top 10%. Memes were randomly but equally selected from the 12 categories thereby created, with the aim of avoiding over-representation of the “peak” time period and meme which received very little attention (as 54.13% of the memes received less than 10 upvotes—reddit’s equivalent of “likes”—and 38.29% of the memes belonged to the 3 weeks peak period, out of the 17 weeks of data collection).
